# Disability and satisfaction after Rotator Cuff decompression or repair: a sex and gender analysis

**DOI:** 10.1186/1471-2474-12-66

**Published:** 2011-04-01

**Authors:** Helen Razmjou, Aileen M Davis, Susan B Jaglal, Richard Holtby, Robin R Richards

**Affiliations:** 1 Holland Orthopaedic & Arthritic Centre, Sunnybrook Health Sciences Centre, Toronto, Canada; 2 Department of Surgery, Sunnybrook Health Sciences Centre, Toronto, Canada; 3 Department of Surgery, University of Toronto, Toronto, Canada; 4 Department of Physical Therapy, University of Toronto, Toronto, Canada; 5 Graduate Departments of Rehabilitation Science and Health Policy, Management and Evaluation, University of Toronto, Toronto, Canada; 6 Division of Health Care and Outcomes Research and the Arthritis Community Evaluation Unit, Toronto Western Research Institute, Toronto, Canada

## Abstract

**Background:**

Rotator-cuff pathology is the most common cause of pain and disability in the shoulder. Examining the combined effect of biological and societal factors on disability would potentially identify existing differences between men and women with rotator cuff pathology which would help to provide suggestions for better models of care. Purpose of this study was to determine the overall differences in disability between men and women and to examine the relationship between factors that represent sex (biological factors) and gender (non-biological factors) with disability and satisfaction with surgical outcome 6 months after rotator cuff surgery.

**Methods:**

Patients with impingement syndrome and/or rotator cuff tear who underwent rotator cuff surgery completed the Western Ontario Rotator Cuff (WORC) index, the American Shoulder & Elbow Surgeons (ASES) assessment form, and the Quick Disabilities of the Arm, Shoulder and Hand (QuickDASH) outcome measures prior to surgery and 6 months post-operatively. They also rated their satisfaction with surgery at their follow-up appointment.

**Results and Discussion:**

One hundred and seventy patients entered into the study (85 men and 85 women). One hundred and sixty patients (94%) completed the 6-month assessment. Women reported more disability both prior to and after surgery. Disability at 6 months was associated with pain-limited range of motion, participation limitation, age and strength. Satisfaction with surgery was associated with level of reported disability, expectations for improved pain, pain-limited range of motion and strength.

**Conclusions:**

The results of this study indicate that women with rotator cuff pathology suffer from higher levels of pre- and post-operative disability and sex and gender qualities contribute to these differences. Gender-sensitive approach will help to identify existing differences between men and women which will help to promote more effective and tailored care by health professionals.

## Background

Sex is the biological characteristics such as anatomy and physiology that distinguish males and females. Gender refers to socially constructed roles and relationships, personality traits, attitudes, behaviors, values, relative power and influence that society ascribes to two sexes based on a differential basis [1]. While sex is a universal condition of humans, gender roles vary across cultures [2-5].

Women and men have different experiences, behaviors and expectations related to their different perception of responsibilities for family and society. These experiences are accompanied by different health risks and different health behaviors, which lead to different demands for help and a different use of medical health services [6,7]. To reduce gender disparities in health and to improve women's access to health services, sex and gender differences need to be studied comprehensively [8]. Unfortunately, the terms "sex" and "gender" are often used interchangeably in the literature with gender usually used as a synonym for sex. Some examples in the field of orthopaedics are related to using the term gender when examining physical and biological attributes, such as muscle mass, bone density, tendon or ligament hypermobility. In addition to inappropriate use of the term "gender", there is no consensus on the proper term when the cause of difference between men and women is due to an interaction between biological and non- biological factors. This complex interplay can create difficulty in shaping conceptual and statistical models which may affect clinical practice.

Neck and shoulder disorders are reported to be more common among women than men in the general population [9]. Among shoulder problems, rotator cuff pathology is the most common condition for which patients seek treatment [10]. Symptoms related to rotator cuff pathology affect one's perception of health [11-15] and account for more than 4.5 million visits with clinicians and approximately 40,000 surgeries per year in the United States [16]. Recovery from rotator cuff surgery has been a subject of investigation since 1923 [17] and the literature indicates that both open and arthroscopic rotator cuff repairs reliably improve functional deficits and pain [18-21]. However, the previous literature does not provide consistent information on the impact of patient's sex on recovery from rotator cuff surgery [22-25]. In addition, inappropriate substitution of "gender" for "sex", using unequal number of men and women for analysis and lack of adjustment for confounding factors limit the interpretation of the results of the literature in this area.

The objectives of this study were:

1. To determine the overall differences between men and women prior to and 6 months after rotator cuff surgery,

2. to investigate the role of sex and gender in perceived disability 6 months after surgery,

3. to determine the role of sex and gender on satisfaction 6 months post-operatively.

The corresponding hypotheses were:

1. Women would have higher levels of co-morbidity and pathology prior to surgery. Women would have more restriction in social activities and more limitation in strength and range of motion both prior to and 6 months after surgery. Women would report higher levels of disability before and after surgery,

2. sex and gender-related factors would affect disability reported 6 months post-operatively,

3. finally, it was hypothesized that sex and gender-related factors would independently predict satisfaction with surgery at 6 months post-operatively.

The explanation on what represents sex, gender, and sex/gender interaction is provided in the section of operational terms.

## Methods

### Subjects

The present study was a prospective longitudinal study of patients with tendonitis, subacromial impingement or full thickness tears of the rotator cuff who required rotator cuff surgery. The target sample was patients referred to one of two surgeons with subspecialty interest in shoulder and upper extremity reconstruction surgery in a large academic institution. The inclusion criteria included age ≥18 years, a diagnosis of impingement syndrome and/or rotator cuff disease, and unremitting pain in the affected shoulder that had not responded to conservative treatment (oral medication, physical therapy, or subacromial injection) for at least 6 months since onset. The diagnosis was based on clinical and radiological findings. The impingement syndrome was defined as an inflammation of the rotator cuff tendons, and/or subacromial-subdeltoid bursa under the coracoacromial arch. Partial thickness and full-thickness cuff tears were defined as incomplete or complete tear of the tendon(s). The exclusion criteria included inability to speak or read English, previous shoulder surgery on the affected side, evidence of major joint trauma causing fracture, infection, underlying metabolic or inflammatory disease, avascular necrosis, frozen shoulder, major medical illness, and psychiatric illness that precluded informed consent. To exclude outliers that could affect the integrity of the analysis, patients with massive rotator cuff tears with or without arthropathy (massive tears with lack of contour and superior migration of humeral head, and severe erosion of glenoid and undersurface of the acromioclavicular joint) [26] were excluded from the study intra-operatively. An informed consent was provided and the rights of the subjects were protected. Approval for use of human subjects was obtained from the research ethics board of the Sunnybrook Health Sciences Centre and University of Toronto.

### Operational terms

To differentiate between terms sex and gender, in the present study "sex" referred to physiological factors where "gender" referred to non-biological factors. Although, it would be optimal to provide distinctions between all factors that represent sex and gender, disentangling sex from gender may not be always possible and therefore more complex factors were examined as "sex/gender" interactions.

The following section describes the classification of factors examined in the present study. **Sex factors: ***Strength *is a biological and physiological property of muscles. Strength may be influenced by non-biological factors such as diet and exercise. However, in the non-athlete sample included in this study, it was felt that strength was more reflective of biological differences. **Gender factors: **Participation limitation and expectation for recovery were classified as gender-related factors. *Participation limitation *is defined as a problem an individual may experience in involvement in life situations [27]. Participation in social activities or roles is affected by society and environment and therefore, it was examined as a "gender" variable. Similarly *expectation for recovery *is affected by previous experiences and culture and is representative of gender. **Sex/Gender factors: **The following factors were examined as a product of an interaction between "sex and gender": Aging, active range of motion limited by pain, Body Mass Index (BMI), extent of comorbidity, severity of bony and soft tissue pathology, medication use, work status, and overall perceived disability. *Age *is a complex factor. We cannot simply extract the biological components of aging from its social components. Hormonal, biological, social, economical, and cultural differences affect the overall aging process and life expectancy [28-32]. *Perception of pain severity *is partly related to difference in neural and hormonal function [33,34] and partly related to social conditioning and cultural upbringing [35]. Similarly, *medication use *is affected by pain sensitivity, drug dependency traits, negative affect and other psychosocial factors. In the present study, *work status *was examined in relation to having a work-related injury (yes/no) and was examined as a sex/gender interaction factor. The literature indicates poorer surgical outcome in patients with work-related injuries due to physical and psychological factors [36-43]. Therefore, sustaining an injury at work may be related to biological factors (i.e. weakness, impaired balance, vision changes, associated illness, etc.) and non-biological factors (i.e. exhibiting behaviors that increase risk of sustaining injury, work load, education, medication use, inactivity, inadequate diet, and etc.). The *BMI*, *co-morbidity*, and *extent of pathology *are affected by complex sex and gender interaction as well. The average height for each sex is significantly different, with adult males usually being taller than adult females [44]. This difference is attributed to sex chromosomal and hormonal differences [45] which together may increase susceptibility to injury in women. Apart from sex related differences in height and weight, gender related factors such as lifestyle, nutrition, sleep patterns, and physical labor can affect the body size, BMI, comorbidity, and susceptibility to developing shoulder problems. Moreover, roles and responsibilities and differences in risk taking behaviors and pattern of health utilization [7,46-48] affect the above factors and should be taken into consideration.

### Outcome Measures of Disability

In this study we used a *disease-specific *measure, a *joint-specific *measure, and an *upper extremity-specific *measure to provide more evidence that rotator cuff related symptoms and functional difficulties affect women more significantly regardless of the focus of the instrument. The primary outcome of disability was the Western Ontario Rotator Cuff (WORC) index [49], a disease-specific measure that explores the impact of rotator cuff disease on different aspects of well-being. The WORC consists of 21 items, each with a visual analogue scale type response option. This measure has five domains: physical symptoms (600 points), sports and recreation (400 points), work (400 points), life style and social function (400 points) and, emotions (300 points). The highest or most symptomatic total score of the WORC is 0% and the highest functional status level is 100%. The WORC has been reported to be reliable and valid [37,50-52] in patients with rotator cuff disease. The ASES is a 100-point scale, 50 points of which are derived from patient self-report of pain on a visual analog scale and 50 points of which are computed from a formula using the cumulative score of 10 activities of daily living derived using a four-point ordinal scale. Activities of daily living include skills such as putting on a coat, sleeping on the affected side, and combing one's hair. The American Shoulder & Elbow Surgeons (ASES) has been reported to be reliable [53] and valid [51,52,54] in patients with shoulder and rotator cuff pathology. The *Quick *Disabilities of the Arm, Shoulder and Hand (*Quick*DASH) is an upper-extremity disability measure [55,56]. The disability/symptom module of this questionnaire has 11-items. The scores range from 0 to 100 with higher scores indicating higher disability. The *Quick*DASH has been reported to have discriminant validity in patients with rotator cuff pathology and shoulder problems [51,57]. To avoid multiple comparisons which increase the change of false positives, the secondary outcomes (ASES, *Quick*DASH) were used only to answer the first objective which investigated the overall differences between men and women in disability. All measures were collected prior to and 6 months after surgery.

### Demographics and Clinical History-Related Factors (Pre-operative data)

The difference between men and women was examined in the following; hand dominance, affected side, symptom duration and characteristics, work status, medication use related to the affected shoulder, smoking habits, and mechanism of injury. Demographic data that were examined in relation with disability included age, BMI, and comorbidity. The comorbidity score was calculated as continuous data based on the validated Cumulative Illness Rating Scale [58]. Thirteen systems were assessed with zero representing no impairment and 4 representing the highest level of possible impairment in that system. The total score ranged from 0 to 52.

### Expectations, participation limitation, and satisfaction with surgery

Patients' expectations for recovery were documented prior to surgery. The expectation questionnaire included seven questions relating to pain relief, range of motion, activities of daily living, work, sports or leisure activities, interacting and providing care for others and overall expectation for recovery following surgery. Participants responded on a 4-5 point Likert scale. This questionnaire has established reliability [51] and validity [37,59] in patients with rotator cuff pathology and knee arthritis.

The extent of participation limitation at 6 months post-operatively was measured by one of the disability questions of the *Quick*DASH: the interference of the upper extremity problems with participation in family/social activities was recorded on a 5-point Likert scale. Categories related to participation restriction were then collapsed into three categories of low, moderate and high.

All patients were asked the following question 6 months post-operatively: "How satisfied are you with the results of your surgery?" The satisfaction level was rated on a 4-point Likert scale; "very satisfied, somewhat satisfied, a little bit satisfied, and dissatisfied".

### Clinical, Radiological and Surgical Factors

#### Clinical Factors

Pre and post-operative clinical examination of the affected shoulder included active pain limited range of motion (ROM). The pain-limited range of motion was examined in 4 directions of flexion, abduction, external and internal rotation (range of 0 to 40 points, with 0 being the most restricted and 40 being the full score) [60]. The European Society for Surgery of the Shoulder and Elbow (ESSSE) recommends pain-limited ROM as a better representation of function in patients with shoulder disorders [61]. Strength measurement in the scapular plane and 90 degrees of elevation was conducted by a simple unsecured tensiometer. The maximum force that the patient could resist for 5 seconds without significant pain and discomfort as the examiner pulled down on the device was measured. One point was awarded for each pound lifted. In case of severe pain while holding the position, strength was given a score of zero, the lowest possible score with no limit for the highest score. The details of clinical assessment and scoring of the pain-limited ROM and strength have been previously published [54].

#### Radiological Factors

The extent of bony pathology was examined radiologically prior to surgery. The information on existence of subacromial spurs, superior migration of humeral head, calcified tendinitis, osacromiale, and degenerative changes in the acromioclavicular (AC) and glenohumeral joints was taken from the radiologist's report. Existing pathological features in the report were recorded as 'yes' in the extraction data collection form, while normal findings were recorded as 'no'.

#### Intra-operative Factors

All patients underwent open or arthroscopic procedures based on the surgeon's preference (two surgeons were involved in this study). Patients with tendonitis, impingement syndrome or partial thickness tears of the rotator cuff tendons underwent arthroscopic or open decompression. Acromioplasty involved ligament release and partial resection as indicated if impingement was caused by anterior acromial osteophytes or coracoacromial ligament thickening. Pathology in the AC joint was managed by removal of the osteophyte or resection of lateral clavicle as indicated.

Patients with full thickness tears of the rotator cuff underwent arthroscopic or open repair of the tendon(s). Repairs were performed for tears involving more than fifty percent of the thickness of the tendon or if there was a significant flap potentially causing mechanical symptoms. Size of rotator cuff tear (largest dimension) was categorized intra-operatively as small < 1 cm, moderate (1-3 cm.), large (> 3-5 cm.), and massive (> 5 cm.)[62]. Low grade partial tears of biceps (≤ 50%) were debrided. Tenodesis was performed for high-grade partial tears (> 50%) of the tendon.

#### Post-operative Care

Rehabilitation was based on the type of surgery. Passive and active assisted motion of the shoulder was initiated on day 1 post-decompression. Active exercises were implemented on day 3 post-operatively. By post-operative day 7, strength exercises were encouraged. Early passive and active assisted motion of the shoulder was initiated on day 1 post-operatively following open rotator cuff surgery. Similar exercises were initiated at 4 weeks post-op following arthroscopic repair. At 6 weeks post-op, sub-maximal isometric exercises started and patients were encouraged to use the affected arm for gentle activities of daily living in front of the body. Strength exercises against resistance were delayed until 12 weeks post surgery for both open and arthroscopic repair. A standardized rehabilitation program was given to patients following surgery to be performed under supervision of their physiotherapist. However the degree of adherence to program was not monitored.

### Follow-Up Assessment

The follow-up visit was 6 months (± 2 weeks) post-operatively. Patients who were unable to attend their 6-month visit were sent a complete set of questionnaires via mail or were contacted by telephone. The frequency of surgical and clinical complications was recorded for infection, rotator cuff rupture, deltoid dysfunction, and nerve injury (axillary, musculocutanous, ulnar and radial nerves).

### Gender-Sensitive Analysis

Sample size calculation was based on the primary outcome measure, the WORC. Using pilot data, 170 patients were considered to be sufficient to detect a 12% difference between men and women accounting for 15% loss of follow up [51]. To address the first purpose, we conducted a descriptive analysis according to sex of the patient. T-tests and chi-square tests were performed for continuous and categorical data as appropriate. To address the second and third objectives we conducted univariable, multivariable and subgroup analyses. These analyses examined the relationship between indicators of sex and gender with "disability" and "satisfaction with surgery". *Univariable *analysis was conducted to identify factors that were related to disability or satisfaction. *Multivariable *analysis using ordinary least squares used all variables that were significant in the univariable analysis (at < 0.1) and examined the combined impact of all factors on the post-operative disability. It should be noted that adjusting for sex of the patient in multivariable analysis without examining the impact of such adjustments could lead to faulty conclusions. Such analyses do not permit determination of the specific ways in which health experiences manifest differently in men and women. Silverstein et al [63] have shown that "adjustment" of the factor of sex in multivariable analyses can mask important differences between men and women. Moerman and van Mens-Verhulst, [64] explain that "in a multivariate model that combines data on women and men, the variable sex may initially display a significant relationship with the health outcome that weakens or even disappears after adding other variables. What actually happens is that the interplay of the factors comprised in the variable sex is decomposed into separate components of biological, psychological or social origin, which play a role in the health problem under study". Therefore, statistical insignificance of the sex factor should not be interpreted as lack of difference between men and women. The insignificance implies that qualities that are hidden in this variable are decomposed to other variables that represent sex and gender. The final step involved subgroup analysis to evaluate heterogeneity according to sex of the patient and generate hypothesis. Assumptions of normality and independence of the residuals (Durbin-Watson statistics), linearity of the dependent and each interval level independent variable, and homoscedasticity (Levene's test of homogeneity of variance) were examined. Multicollinearity among the independent variables was assessed. Plausible interactions, particularly factors that were different in subgroup analysis were examined among variables prior to proceeding with multivariable analysis. Statistical analysis was performed using SAS^® ^version 9.1.3 (SAS^® ^Institute, Cary, NC). Statistical results are reported using 2-tailed p values with significance set at p < 0.05.

## Results

Due to the larger number of male candidates for surgery and the need to maintain an equal number of men and women in the study we continued recruiting women after sufficient and equal number of subjects had been included in each sex group. One hundred and eighty five (91 females and 94 males) patients were recruited into the study. Fifteen patients were excluded intra-operatively. Seven patients had massive rotator cuff tears and 8 patients had arthropathy. Therefore, 170 patients were included in the study (85 men and 85 women). There were no surgical complications. One hundred and sixty patients (94%) completed the 6-month assessment (78 females and 82 males; mean age; 57 ± 11, range: 32-87). Out of these 160 patients, 8 patients (4 females and 4 males) did not physically attend their follow-up appointment and completed the subjective measures via mail or telephone. The means of the WORC, strength, and range of motion of the initial cohort were 35 ± 20, 5 ± 3, and 22 ± 10 respectively. The means of the WORC, strength, and range of motion of the patients who completed the study were 38 ± 17, 5 ± 4, and 21 ± 10 respectively. As such, there was no statistically significant difference between all study patients and those who completed the study (p > 0.05).

### Overall differences between men and women

Women were statistically significantly older and had a slightly higher level of comorbidity. Pre-operative BMI, level of rotator cuff pathology, biceps pathology, associated osteoarthritis, symptom duration, symptom characteristics, and medication taken pre-operatively were not significantly different between men and women. However, once BMI was categorized as normal, overweight and obese, women fell more in the obese category where men fell in the overweight category. The number of women who had a rotator cuff repair or biceps tenodesis was not statistically different than men. The tear size of the rotator cuff was not statistically significantly different between men and women who had a repair (Table 1). The objective measures of pain-limited range of motion and strength were also statistically significantly lower in women both prior and after surgery. Women reported more participation restrictions on both time points (Table 2). Women reported more disability as documented by all subjective outcome measures both prior and after surgery (Table 3).

**Table 1:** Demographic information of the final cohort

Variables	Women (%) N = 78	Men (%) N = 82	Statistics P values
Age	59 (10.64)	55 (11.28)	t value:2.30, p = 0.0228

Level of pathology			
Number of repairs	42	49	χ²: 0.57, p = 0.4506
Number of decompressions	36	33	

Biceps tenodesis	3	8	Fisher's Exact Test:0.09, p = 0.21

Associated Osteoarthritis	47 (60%)	46 (56%)	χ²: 0.28, p = 0.5940

Work status (Active Compensation) Yes/no	19/59	15/67	χ²: 0.88, p = 0.35

BMI (Mean, SD)	30 (7)	29 (5)	t value = 0.72, p = 0.47
• Normal < 25	20 (26%)	11 (13%)	χ²: 8.49, p = 0.014
• Overweight (25.0-29.99)	24 (31%)	43 (52%)	
• Obese (≥ 30.0)	34 (44%)	28 (34%)	

Comorbidity (range: 0-52)	3.48 (2.91)	2.02 (2.44)	t value: 2.17, p = 0.03

Smoking			
• Yes	7 (9%)	15 (18%)	χ²: 2.93, p = 0.09
• No	71(91%)	67 (82%)	

Hand Dominance			
• Right	71 (91%)	74 (90%)	Fisher's Exact Test: 0.11,
• Left	7 (9%)	7 (9%)	p = 1.00
• Bilateral	None	1 (1%)	

Affected Side			
• Right	45 (57%)	40 (49%)	χ²: 5.19, p = 0.074
• Left	12 (15%)	25 (30%)	
• Bilateral	21 (27%)	17 (21%)	

Side operated on			
• Right	57 (73%)	48 (59%)	
• Left	21 (27%)	34 (41%)	χ²: 3.15, p = 0.05

Symptom duration in months (Mean, SD)	43.42(84)	46.48 (59)	t value: -0.29, p = 0.77

Symptoms characteristics			
• Pain on movement	60 (77%)	67(79%)	χ²: 0.13, p = 0.85
• Night pain	54 (69%)	49(60%)	χ²: 1.56, p = 0.21
• Weakness	52 (67%)	55 (67%)	χ²: 0.03, p = 0.96
• Catching/Clicking/Grinding	37 (47%)	39 (48%)	χ²: 0.0003, p = 0.99

Mechanism of injury			
• Insidious	24 (31%)	23(28%)	χ²: 0.14, p = 0.71
• Repetitive activities	14(18%)	13 (16%)	χ²: 0.13, p = 0.72
• Fall	15 (19%)	10 (12%)	χ²: 1.50, p = 0.22
• Traumatic	9 (12%)	19 (23%)	χ²: 3.74, p = 0.05

**Table 2:** Differences between men and women in objective measures and participation limitation prior and after surgery

Variables	Women, N: 78 Mean (SD)	Men, N: 82 Mean (SD)	Statistics P values	Effect size (CI)
*PRE-OPERATIVE*				

Strength*				
(Elevation in scapular plane, lb)	2.9 (2)	6.95	-5.72	1.01
			< 0.0001	0.68-1.35

Pain limited Range of Motion*				
(measured in 4 directions, 0-40)	19 (9.7)	23(10.0)	-2.55	0.40
			0.01	0.08-0.71

Participation limitation				
• Low	29(37%)	43(52%)	χ²: 11.20,	_______
• Moderate	17(22%)	25(30%)	p = 0.004	
• High	32(41%)	14(17%)		

*POST-OPERATIVE*				

Strength*				
(Elevation in scapular plane, lb)	5.6 (3.5)	13(5.2)	-8.33	1.78
			< 0.0001	1.40-2.16

Pain limited Range of Motion*				
(measured in 4 directions, 0-40)	28(11)	35(6)	-4.65	0.72
			< 0.0001	0.40-1.05

Participation limitation				
• Low	49(65%)	69(85%)	χ²: 10.60,	_______
• Moderate	10(13%)	8(10%)	p = 0.005	
• High	16(21%)	4(5%)		

T Test (T values): used for normally distributed data

*Wilcoxon-Mann-Whitney test (Z values): used for skewed data

χ²: Chi-Square

**Table 3:** Pre and post-operative differences in disability level

Variables (Min/Max)	Women Mean (SD)	Mean (SD)	Statistics P values
*PRE-OPERATIVE*			

WORC (0/100%)	34.92 (18)	41.48 (17)	-2.40 0.018
Symptoms (0/600)	351.03(130)	324.68 (109)	1.38 0.1697
Life style* (0/400)	269.73 (88)	219.57 (82)	3.72 0.0003
Work* (0/400)	286.12 (69)	247.98 (79)	3.26 0.0014
Sports/recreations (0/400)	289.83 (73)	281.70 (67)	0.73 0.4669
Emotions (0/300)	169.65 (75)	154.84 (78)	1.22 0 .2225

ASES (0/100)	42.60 (22)	51.24 (16)	-2.83 0.0053

*Quick *DASH (0/100)	55.50 (19)	44.60 (17)	3.79 0.0002

*POST-OPERATIVE*			

WORC (0/100%)	60% (26)	74% (20)	-3.34 0.0008
Physical Symptoms* (0/600)	196 (145)	141(112)	2.21 0.0273
Life style* (0/400)	152 (117)	85 (85)	3.74 0.0002
Work* (0/400)	188 (119)	110 (100)	4.17 < 0.0001
Sports/recreations (0/400)	194 (113)	132 (90)	3.50 0.0005
Emotions* (0/300)	104 (100)	69 (82)	2.24 0.026

ASES* (0/100)	69 (22)	79 (19)	-3.066 0.002

*Quick *DASH* (0/100)	35 (24)	19 (18)	4.52 < 0.0001

T Test (T values): used for normally distributed data

*Wilcoxon-Mann-Whitney test (Z values): used for skewed data

### Analysis of Disability

The univariable ordinary least squares analysis showed that the WORC score at 6 months was associated with sex of the patient, age, pre-op level of WORC score, post-op strength and range of motion, participation restrictions, level of pathology (type of surgery), work status, medication use, and pre-operative expectations for return to work (Table 4). The exploratory subgroup analyses showed consistency between the overall effect and the differential subgroup effect with a similar pattern of relationship between disability and participation limitation, strength, work status, and pain limited range of motion (Table 5). Age was associated with post-op disability in women only with younger women feeling more disabled than older women. Men appeared to have a more homogenous level of disability at 6 months which was not necessarily related to their pre-op disability (SD: 20). Women had more variability in their post-op disability (SD: 26) which was more consistent with their pre-op level of disability (17% of variance of post-op WORC was explained by the pre-op WORC in women vs. 3% in men). Medication use and expectations for return to work had more variability in men. Men who were taking medication before surgery reported higher disability indicating that taking medication prior to surgery is a more negative predictor of post-op disability in men. To examine the vigor of subgroup analysis of factors that were not consistent with the overall effect in the "entire sample", we examined the interactions between sex and these factors and found no interactions between sex of the patient and age (F = 0.33, p = 0.57), sex and pre-operative disability (F = 3.48, p = 0.06), or sex and medication use (F = 1.39, p = 0.24). Therefore, the above trends in each sex group should be interpreted with caution.

**Table 4:** Univariable analysis of disability

Independent variables	DF (R-square)	F value	P value
Binominal factor of man/woman	1 (0.08)	14.58	0.0002

Age	1 (0.03)	4.33	0.0390

Pre-operative WORC	1 (0.12)	21.33	< 0.0001

Post-op pain limited ROM	1 (0.54)	178.50	< 0.0001

Comorbidity	1 (0.0008)	0.13	0.7177

BMI	1 (0.0003)	0.06	0.8089

Post-op strength	1 (0.31)	68.41	< 0.0001

Post-op participation limitation	2 (0.53)	4.33	0.006

Pre-op expectations for return to work	3 (0.08)	2.81	0.0422

Work Status	1 (0.14)	25.50	< 0.0001

Level of pathology	1 (0.03)	5.22	0.0237

Pre-op Medication use	1 (0.03)	5.61	0.0191

**Table 5:** Subgroup analysis of disability

Independent variables	DF (R-square)	F value	P value
WOMEN			

Age	1 (0.06)	5.03	0.0279

Pre-operative WORC	1 (0.17)	15.36	0.0002

Post-op pain limited ROM	1 (0.62)	117.60	< 0.0001

Post-op strength	1 (0.30)	30.72	< 0.0001

Post-op participation limitation	2 (0.56)	46.44	< 0.0001

Pre-op expectations for return to work	3 (0.03)	0.76	0.5184

Work Status	1 (0.16)	14.15	0.0003

Level of pathology	1 (0.04)	3.00	0.0872

Pre-op Medication use	1 (0.003)	0.20	0.6527

MEN			

Age	1 (0.04)	3.54	0.0637

Pre-operative WORC	1 (0.03)	2.70	0.1045

Post-op pain limited ROM	1 (0.25)	25.89	< 0.0001

Post-op strength	1 (0.20)	19.01	< 0.0001

Post-op participation limitation	2 (0.41)	26.71	< 0.0001

Pre-op expectations for return to work	3 (0.20)	6.29	0.0007

Work Status	1 (0.11)	9.92	0.0023

Level of pathology	1 (0.01)	1.58	0.2122

Pre-op Medication use	1 (0.08)	7.36	0.0081

In the multivariable regression analysis that examined all significant variables in one model, 73% of the disability variance was explained by 3 factors: 1) participation limitation (less limitation was associated with lower disability), 2) range of motion and strength (higher strength and better range of motion were associated with lower disability) and 3) age (older patients reported less disability). The binominal factor that represented all man and woman qualities lost its significance in the multivariable analysis as it decomposed into sex, gender, and sex/gender qualities (Table 6). In summary, disability was affected by sex differences (i.e. strength), gender differences (i.e. participation limitations), and factors that represented sex and gender interaction (i.e. age, pain-limited range of motion).

**Table 6:** Multivariable analysis of disability

Independent variables	Estimate	DF (R square)	F value	P value
Factor of man/woman	Female: -0.60 Male: 0.00	1	0.03	0.8554

Age	0.32	1	5.81	0.0173

Pre-operative WORC	-0.061	1	0.65	0.4234

Post-op pain limited ROM	0.90	1	26.87	< 0.0001

Post-op strength	0.67	1	5.53	0.0202

Post-op Participation limitation	1: 27.62	2	23.40	< 0.0001
	2: 10.14			
	3: 0.00			

Pre-op expectations for return to work	Working:7.06	3	1.89	0.1349
	N/A: 5.98			
	Light:1.07			
	Full:0.00			

Work Status	Yes: -2.33	1	0.27	0.6069
	No: 0.00			

Level of pathology	Tear: 0.52	1	0.10	0.7571
	No tear: 0.00			

Pre-Medication use	Yes: -1.25	1	0.41	0.5243
	No: 0.00			

FULL MODEL		13 (0.73)	27.38	< 0.0001

### Analysis of Satisfaction

The univariable ordinal logistic regression analysis showed that factors that represented sex (i.e. strength), gender (i.e. expectations and participation limitation) and sex/gender interaction (i.e. pain limited ROM, disability, work status) had statistically significant associations with satisfaction. Subgroup analysis (Table 7) revealed that women with less strength reported less satisfaction with surgery which was not the case in men. The other inconsistency between men and women was the impact of work-related injury on satisfaction. No relationship was observed in men while women who were receiving benefits from the compensation board reported less satisfaction than women who were not involved in work-related injuries. The multivariable ordinal logistic regression met the assumptions of proportional odds and lack of multicollinearity. Factors that remained significant in the multivariable model were disability, expectations for pain relief, range of motion and strength (Table 8). Logically, more limitation in range of motion and strength at 6 month was correlated with less satisfaction with surgery. Patients who had higher levels of expectation for pain relief reported less satisfaction.

**Table 7:** Subgroup logistic ordinal regression analysis of satisfaction

Variables	Estimate	Odd Ratio (CI)	Wald Chi-square	P value
WOMEN				

Post-op WORC	0.10	1.11(1.07-1.14)	34.56	< 0.0001

Post-op Pain limited ROM	0.16	1.18(1.11-1.25)	29.89	< 0.0001

Post-op Strength	0.23	1.26(1.09-1.45)	9.73	0.0018

Post-op Participation restriction				
• Low	1.58	21.57(5.94-78.25)	19.42	< 0.0001
• Moderate	-0.09	4.06(0.87-18.93)	0.043	0.8364
• High	0.00			

Work Status	-0.64	0.28(0.10-0.75)	6.40	0.0114

MEN				

Post-op WORC	0.08	1.09(1.05-1.12)	28.23	< 0.0001

Post-op Pain limited ROM	0.15	1.16(1.07-1.25)	13.64	0.0002

Post-op Strength	0.075	1.08(0.98-1.18)	2.63	0.1049

Post-op Participation restriction				
• Low	1.73	14.29(1.89-13.57)	13.57	0.0002
• Moderate	-0.80	1.13(0.11-11.05)	2.04	0.1531
• High	0.00			

Work Status	-0.09	0.84(0.28-2.51)	0.10	0.7553

**Table 8:** Multivariable logistic ordinal regression analysis of satisfaction

Independent Variables	Estimate	Odd Ratio (CI)	Chi Square	P value
Factor of man/woman				
Female	0.22	1.56(0.52-4.69)	0.62	0.43
Male	0.00			

WORC	0.08	1.09(1.05-1.12)	24.35	< 0.0001

Pain limited ROM	0.08	1.08(1.01-1.15)	5.55	0.0184

Strength	0.11	0.89(0.80-0.99)	4.23	0.0397

Expectations for improved pain				
High (a lot)	-0.69	0.25(0.08-0.75)	6.07	0.0138
Moderate (a bit/somewhat)	0.00			

Participation limitations				
Low	0.27	1.79(0.37-8.59)	0.45	0.50
Moderate	0.05	1.44(0.29-7.00)	0.01	0.91
high	0.00			

Work Status				
Positive work-related injury	0.17	1.40(0.49-4.01)	0.30	0.53
Negative work-related injury	0.00			

## Discussion

Investigating the biological and social/cultural factors in musculoskeletal health will help to improve our knowledge about etiology and will result in more effective interventions. This study demonstrates that a number of biological and non-biological factors affect disability and satisfaction 6 months following rotator cuff surgery. Descriptive data indicated that women included in this study were more disabled both prior to and after surgery despite having similar levels of bony and soft tissue pathology. They were more limited in their strength, range of motion and participation in family and society-related activities.

Previous literature indicates an overall difference in disability and response to treatment between men and women. Oh et al [25]who examined recovery following a rotator cuff repair at a minimum of one year reported that the female patients had an inferior response to treatment as measured by the Simple Shoulder Test (SST), a subjective disability questionnaire. O'Holleran et al [22]reported that patient's sex (95 women and 216 men) was not a significant determinant of improvement after surgery, while Romeo et al[24] and Charousset et al [23] reported lower improvement in women undergoing rotator cuff surgery. Romeo et al. [24] examined 28 women and 44 men with full-thickness tears and found that disability as defined by the Constant Murley score (CMS) and SST had a small negative correlation with age in women but not in men. Charousset et al [23] who examined 104 patients (61 women and 53 men) reported statistically significant differences between men and women (female sex being a negative predictor) as measured by the CMS at minimum of 2 years post-operatively. Overall, previous studies do not provide conclusive results due to adjusting for the sex factor without examining the implications, the retrospective nature of the study and small or unequal sample sizes [24] or the fact that investigating these differences was not the primary research question of the study [23].

### Analysis of Disability

In our study, the univariable regression analysis showed that factors that represented sex (i.e. strength), gender (i.e. expectations and participation limitation) and sex/gender interaction (i.e. pain limited ROM, baseline disability, work status, medication use, extent of pathology) had statistically significant associations with post-operative disability. The subgroup analysis showed that age and pre-op level of disability had a different impact on post-op disability in women. Age showed a statistically significant relationship with disability in women. In men, there was a trend towards a similar pattern with older individuals reporting less disability in both sexes. The difference in pre-op disability is more of interest. Women had more variability in their post-op disability which was more consistent with their pre-op level of disability. Therefore, it is more likely that a highly disabled woman would continue to report higher disability 6 months after surgery, whereas pre-op disability is not predictive of post-op disability in men. On the other hand, taking medication was a predictor of poor recovery in men and not in women. These differences may have implications in terms of providing a more tailored treatment program to patients that may potentially have poor recovery based on their pre-operative characteristics. Women with higher disability scores of the WORC and men who are taking medication prior to surgery may benefit from multidisciplinary consultations in terms of coping with their post-operative symptoms and dysfunction. In the multivariable analysis, objective findings that represented sex (strength) and an interplay of sex/gender (pain limited ROM) had a significant impact on how patients rated their residual disability 6 months following surgery. A number of investigators have reported that pre-operative level of range of motion and strength were important indicators of recovery [65-67] but some investigators have not found a significant relationship between these factors and improvement following surgery [68]. Participation restriction remained a strong predictor of disability regardless of other factors in the model supporting the importance given to this component of disability by the World Health Organization [27]. To date, there are no published data on the relationship between disability secondary to rotator cuff disease and its impact on fulfilling family/society roles. Among factors that represent the interplay of sex/gender, "age" had an interesting relationship with disability. Patient's age has been reported to have a negative or insignificant impact on disability [18,20,21,69]. In contrast, some literature indicates a reversed relationship with reported disability or satisfaction following surgery with older patients reporting better function [70-73]. Smaller tears and better cuff integrity in younger patients is expected to be associated with better healing. However, older individuals have lower demands and expectations, which may help them cope better with their rotator cuff problems. Considering rotator cuff pathology involves patients with a wide range of ages, it is likely that younger patients with more physical and emotional demands suffer from higher levels of disability.

### Analysis of Satisfaction

The use of patient satisfaction ratings helps to incorporate patient perspectives into the design of health care services, types of surgical procedures, and overall quality assessment. Understanding how sex differences and gender inequalities affect the overall outcome and satisfaction with surgery would further help to direct the needed services to men and women suffering from disability secondary to shoulder conditions.

The present study shows a relationship between satisfaction with rotator cuff surgery and biological or sex-related factors and non-biological or gender-related factors. Subgroup analysis revealed that strength and work-related injury were related to satisfaction only in women. The implications of these findings may be for post-operative plans by the clinicians, nurse case managers, and orthopaedic surgeons. By providing a sex-specific rehabilitation program (more emphasis on strength) or having a structured return to work plans following a work-related injury which incorporates biological vulnerability of female patients, the satisfaction level of women may be improved. Working or younger women may also require more support with respect to their family responsibilities as they prepare to return to their full duties at work. In the multivariable analysis four factors affected satisfaction following surgery: strength, pain-limited ROM, expectations for improved pain, and pre-op level of disability. Our findings on relationship between satisfaction and disability are consistent with the previous literature [22,74]. In terms of strength and ROM, the results of our study are consistent with the study by O'Hollern et al [22] who reported a positive relationship between reduced active elevation and strength and reduced satisfaction. In terms of expectations, one previous study [75]examined patient satisfaction using a binary question of yes or no. The authors used a Visual Analogue Scale (VAS) to measure the level of satisfaction in those who responded yes. This study found that marital and working status and having higher expectations for treatment were correlated with a better satisfaction level. Scheier et al [76] propose that optimism (i.e., expecting good outcomes) generally has beneficial effects on recovery after surgery. Although in general terms being optimistic appears to influence outcomes in a more positive way, having unrealistically positive expectations may increase dissatisfaction. Janis [77] reports that if a stressful event produces more suffering than expected, the mood will tend to be negative or dysphoric. If suffering is less than expected, the mood will tend to be positive or euphoric. If a person has overoptimistic expectations, the chance that they are not in line with the complaints that are actually experienced increases, which may subsequently increase the probability of disappointment. In accordance with Janis' theory [77], we found that patients who had lower expectations for pain relief prior to surgery were more satisfied after surgery. Since expectations are generally modifiable through education, establishing realistic expectations and goals may improve patient outcome and overall satisfaction with surgery. Suls and Wan [78] suggest that giving information about potential pain and complaints reduces negative effects and pain reports following treatment. In our study, patients who had higher expectations for pain relief, reported less satisfaction. Therefore, it may be beneficial to inform the candidates for rotator cuff surgery of the possibility of residual pain and discomfort at 6 months post surgery as this information may help to reduce the expectation/satisfaction discrepancy.

In summary, the implications of this study relate to the impact of strength, ROM, expectations, and participation limitations on disability. Women appear to be more disabled both prior to and after rotator cuff surgery regardless of similar levels of pathology they had in comparison with men. Prioritization of females by decreasing the waiting period to see a physical therapist or orthopaedic surgeon might be indicated to reduce disability of female patients with rotator cuff pathology. Strength, a physiological factor was an important contributor to disability and satisfaction. Dissimilarity in pain perception in female patients affected by physiological and social/cultural upbringing also affected their pain-limited ROM. The specific implications of these findings can be incorporated into the rehabilitation programs in order to provide sex-based rehabilitation that accounts for women's unique structural and biological differences. In addition participation in family/social activities had a significant impact on perception of disability secondary to rotator cuff pathology both before and after surgery. Women's unique care-giving roles in family and society make them more susceptible to disability as they need to fulfill more responsibilities and expectations compared to their male counterparts. Female caregivers and those who provide care to small children or older individuals at home may benefit from sharing of caregiving responsibilities and having better access to external social resources as they recover from rotator cuff pathologies.

## Limitations

Gender studies are affected by cultural, political, and social factors and therefore our results are applicable to North American men and women with rotator cuff pathology. In the present study despite a large number of factors examined, certain important gender related differences such as marital status, level of income, access to the health care system, and extent of family and social support were not explored. Given the complexity of the relationship between participation restrictions and disability, more comprehensive and sensitive measures of participation are needed to capture the important aspects of involvement in life situations. In the present study, adherence to the recommended rehabilitation regime which is affected by environmental, physical and psychological factors was not measured. This factor may affect the success of the surgery and needs to be examined in future studies. The present study examined the impact of sex and gender factors on disability 6 months after surgery which is considered a short follow-up. Whether differences between men and women would reduce as a function of more time remains to be studied in future studies with a primary focus on examining sex and gender differences. The results of this study may not be applicable to non-academic or community-based hospitals. Multicentre studies will help to improve the generalizability of the findings.

## Conclusion

The results of this study indicate that disability and satisfaction at 6-month following rotator cuff surgery are affected by biological or sex-related differences and non-biological or gender-related differences. Satisfaction with surgery had a strong relationship with residual disability at 6 months post-operation. Age, baseline disability, expectations, medication use, strength, and having a work-related injury showed different levels of association with disability or satisfaction in women.

There are many challenges in conducting gender-sensitive research. Further conceptual clarification, gender-sensitive methodology, and more sophisticated statistical analyses are needed for better understanding of the complex interactions between sex and gender. Sex and gender should not be studied in isolation or be treated as a confounding factor. Gender influences interpretation of biological differences and biological characteristics affect gender disparity in health. Scientific analyses by sex and gender should be encouraged to optimize clinical management of musculoskeletal problems and particularly rotator cuff disease. By identifying biological and non-biological differences that affect disability and satisfaction with rotator cuff surgery, orthopaedic surgeons, physical therapists, occupational therapist, nurse case managers, and ergonomic assessors would be able to set more specific goals and expectations and achieve faster functional recovery.

## Competing interests

The authors declare that they have no competing interests.

## Authors' contributions

This study was conducted in partial fulfillment of the requirements for the degree of Doctor of Philosophy for HR. HR conceived the idea, wrote the protocol, performed the clinical examination, supervised data collection and entry, conducted the analysis, and drafted the manuscript. AMD and SBJ co-supervised the protocol development, statistical analysis, and edited the manuscript. RH and RRR performed the surgical procedures and provided input on study design, protocol development and the manuscript. RRR was the faculty supervisor of the PhD thesis. All authors have read and approved the final manuscript.

## Pre-publication history

The pre-publication history for this paper can be accessed here:

http://www.biomedcentral.com/1471-2474/12/66/prepub
